# Exploring the Effect of Health on Migrants’ Social Integration in China

**DOI:** 10.3390/ijerph19084729

**Published:** 2022-04-14

**Authors:** Xiang Kang, Mingxi Du, Siqin Wang, Haifeng Du

**Affiliations:** 1 School of Public Policy and Administration, Xi’an Jiaotong University, Xi’an 710049, China; kangxiang9523@stu.xjtu.edu.cn; 2 School of Economics and Finance, Xi’an Jiaotong University, Xi’an 710049, China; wangsiqin119@foxmail.com

**Keywords:** Chinese migrants, social integration, health, social participation

## Abstract

There are 376 million migrants, which account for more than 25% of the population in China according to the newest national demographic census, most of whom are from undeveloped areas to developed urban regions. Migrants’ social integration was one of the most important issues when the country aimed to build an inclusive society. As a form of human capital, the effect of migrants’ health status on social integration has rarely been explored until now, especially empirically. Previous studies have usually ignored health indicators when discussing the determinants of migrants’ social integration, and understanding the role of migrants’ physical health and mental health on their social integration is significant for efforts to ensure inclusive urbanization. For filling this research gap, the China Migrants Dynamic Survey dataset was used to uncover the role of migrants’ health status, including physical health and mental health, in their degree of social integration, and a further comparison of impact was conducted among rural–urban and urban–urban migrants. Through the empirical analysis, our results indicated the following. First, both better physical and mental health lead to higher social integration levels, and a one-point increase in physical and mental health improves the odds of good social integration by 33.27% and 5.98% for belonging and 66.05% and 6.35% for harmony, respectively. Second, health status is equally important for rural–urban and urban–urban migrants’ social integration, and the significant positive effect was consistent across groups, although some other impact factors may exhibit differences. Third, the effect of health status on social integration was moderated by social participation, which was more obvious for mental health than physical health. According to our findings, we discuss the measures to promote migrants’ health status and additional countermeasures to improve their social integration level.

## 1. Introduction

China’s rapid urbanization has concentrated the population in urban regions caused mainly by a large number of migrants from underdeveloped regions [1]. Migrants increasingly move to urban regions for job opportunities and a better life, with approximately 220 million such migrants in 2010 and 376 million in 2020, constituting the largest migration in human history [2,3]. Urban marginalized populations face a series of barriers to integrate into cities, which are not only because of insufficient public services, low income and cultural differences, but also include high health risks due to potential hazards [4,5]. How to make the numerous migrants adapt to cities and improve their social integration level has been a huge challenge for scholars and government [6,7,8]. This is especially true under the background that China’s General Office of the State Council formulated the “Proposal to Promote the Settlement of 100 Million Non-Resident Population in Cities”.

In China, migrants have an irreplaceable role in the country’s economic development, which will continually promote further integration of urban–rural areas for coprosperity. However, migrants are also the most vulnerable group in cities due to the institutional discrimination and urban–rural discrepancies, especially for rural–urban migrants. Inefficient social services, poor living conditions and potential discrimination may affect their health; because of their poor socioeconomic status and limited access to public services, they may be discriminated against by others [9,10,11]. Health is not only an important component of human capital, but it is also associated with people’s happiness and the harmony of society [12,13]. As the World Health Organization (WTO) defined, health is a state of physical, mental and social well-being and not merely the absence of disease and infirmity, which was generally accepted by previous studies [14]. Limited social support and resources in host cities and potential discrimination from local residents may impact migrants’ health status. Additionally, migration may pose other risks for migrants; the most important aspect is that the migration experience is tightly associated with health status, which has been proven in previous studies [15,16,17,18]. Meanwhile, the inefficient basic public services and higher life costs than in their hometown make them more vulnerable to health issues, so health issues are a huge problem for these marginalized groups.

With the large number of migrants living and working in cities, which will further increase in the future, the government and researchers have begun to pay more attention to these marginalized groups. In addition, the Chinese government aimed to ensure so-called “people-centered” urbanization and society, which means that more consideration needs to be given to marginalized groups, such as the large number of migrants in metropolis regions [19,20]. Migrants’ social integration, as an important research field and policy issue, is gradually being discussed in China but trails behind that in Western countries. Social integration can provide economic and cultural benefits, which can also ensure social stability [21]. However, according to previous studies, migrants in cities usually have low social integration levels for various reasons [19,22]. Nowadays, migrants’ settlement intention and their social integration are two research focuses. Settlement intention determines whether migrants will live in cities temporally or permanently. However, social integration is more tightly related to migrants’ subjective perception under the background of “people-centered” urbanization, which is also more important for migrants than whether they decided to live in cities temporarily or permanently. Additionally, according to the study of Lu and Wu, social integration is a key factor for migrants’ willingness to acquire hukou, so we can conclude that social integration is a basic individual demand for migrants [23].

There are two classic theories about migrants’ health, including the “salmon bias” hypothesis and the “health migrant” phenomenon. The “salmon bias” hypothesis, which is usually utilized to explain the “health migrant” paradox, implies that migrants with better health status may have a greater desire to settle down in host cities, and according to other studies, health status also plays a crucial role in other areas, such as individual income, life satisfaction and subjective well-being [24,25,26]. The health status of migrants was an important issue in China, especially in this era with a large scarcity of migration phenomena [27]. In the research on migrants’ settlement intention, there is a famous paradox called the “health migrant” paradox in developed counties: although migrants usually have a relatively poor socioeconomic status, their health status is better than that of natives [28,29]. In other words, social integration is a more fundamental demand for migrants than the settlement decision, and the ultimate goal towards building an inclusive society and urbanization is achieving a high level of social integration between migrants and natives.

To the best of our knowledge, few studies have discussed the effect of health status on the degree of social integration among various categories of migrants. Therefore, this study may provide a deep understanding of the effect of health status on migrants’ social integration and may have policy implications for the government to facilitate social integration and achieve the goal of an inclusive society and urbanization. The rest of this article is as follows: Section 2 and Section 3 provide a review of the concepts of social integration and migrants’ social adaptation and the existing literature about health effects. Section 4 describes the data and empirical methodology. The results are presented in Section 5, and we further explore the health status heterogeneity across kinds of migrants. Then, the moderate effect of social participation is discussed. The last two sections discuss the results and conclude the findings and the contributions of this study.

## 2. Social Integration of Chinese Migrants

Previous studies on the social integration of migrants in China, the largest developing country in the world, initially began because a large number of rural workers were leaving their hometowns and migrating to cities for job opportunities and a better life [30]. One of the most important topics in this research field associated with social integration is the hukou system, which is a unique household registration system in China. This unique institution creates an invisible wall between rural and urban residents because they have different hukou types (urban hukou for local urban residents and rural hukou for rural populations). In this system, it is difficult for migrants to obtain a local urban hukou, which is tightly associated with urban public services. With large-scale migrants from other undeveloped regions pouring into large cities, more migrants without a local urban hukou are not entitled to the same certain privileges as local urban residents, which leads to a two-class urban system [31,32]. The hukou system restricts migrants’ mobility and access to social benefits, such as subsidized housing, high-quality health services and public education [33,34]. This invisible wall leads to social inequality, which may form an obstacle for migrants to obtain a better life in cities, especially in some large cities where the urban hukou is more difficult to obtain than other middle-sized and small cities. Therefore, the hukou system has usually been considered as one of the most significant barriers to migrants integrating into cities [35,36]. At the beginning, to control rural–urban migration in China, the strict hukou system was implemented by the central government in the early 1980s and until economic reform [31]. This invisible wall hindered numerous rural populations working and living in cities. Although the Chinese regional government has made great efforts to break out of this system, the difficulties in metropolises are larger than those in middle-sized and small cities.

To date, existing studies have made some efforts to explore the factors impacting migrants’ social integration to find ways to increase migrants’ social integration level. For example, housing conditions have been discussed by several studies, and these studies indicated that people with better housing conditions may have higher social integration levels than those in poor housing conditions [10,37,38]. Social media use can also facilitate migrants’ social integration, including improving social identity and building social networks [26]. Studies have indicated that health insurance has a positive impact on the degree of social integration, so the insurance participation rate needs to be improved, especially for rural–urban migrants [39]. However, as one of the most important factors associated with individuals migrating to cities, whether health status affects social integration in metropolises has rarely been studied.

When discussing migrants’ social integration, we need to confirm the categories of migrants and measure the degree of social integration. The Chinese population, divided by the hukou system, consists of rural and urban residents, so there are two kinds of migrants in cities: rural–urban and urban–urban migrants [40]. The main difference is their place of origin: rural–urban migrants come from rural regions with a rural hukou, and the others come from other cities. According to Chinese census data, although there have been enormous rural-urban migrants in China, the scale of which sharply increased from the early 21st century and decreased later, the scale of urban-urban migrants has increased steadily [41]. With the population persistently pouring into metropolises or urban agglomeration, the shrinking cities phenomenon may become common in the future, so the urban–urban migrants also need to be considered in the host cities, such as their degree of social integration [42]. It is necessary to distinguish between these groups because of their different living conditions and lifestyles. More importantly, rural–urban migrants’ social integration may be lower than that of urban–urban migrants, which requires additional study [22,37]. Thus, our study provides a new perspective when studying the migrants’ social integration, which is necessary and innovative.

The measurement of social integration is complicated and has no consistent measure. For migrants, social integration is usually defined as a process of fusion between them and natives. According to previous studies, social integration has been divided into subjective social integration and objective social integration from two different perspectives, and subjective social integration is more important and directly originates from social interaction [43]. Research has shown that subjective factors are more significant for subjective well-being than objective factors, and the target of “people-centered” urbanization should be the subjective perception of migrants in cities [26]. Existing literature on the measurement of social integration has usually focused on objective factors, such as income, insurance participation and housing conditions. Utilizing these indicators to represent the degree of social integration is relatively objective. According to the summary of Wang and Fan, three forms of social integration have been primarily adopted by previous studies, with these generally involving economic, social, cultural and identity factors [35]. Economic, social and cultural factors are the objective indicators, and identity is a subjective indicator that is more tightly associated with individuals’ perceptions and subjective happiness. Although objective indicators can show the external condition or requirement of social integration, migrants’ perceptions are difficult to measure through these indicators or related evaluation frameworks. Additionally, it is difficult to confirm the weights of various indicators when building evaluation systems, although different objective indicators obviously have different abilities to represent social integration. Nevertheless, individuals’ perceptions of social integration can play a more direct role in representing the degree of social integration. Therefore, in this study, when referring to the social integration level, we try to represent it from a more subjective perspective that is more appropriate for “people-centered” urbanization and society and for taking migrants’ real perceptions into consideration.

## 3. Health, Migration and Social Integration

Health status is the general performance of individual’s health, and which is usually regarded as the one of the most important human capitals [44,45]. According to the US Center for Disease Control, health status refers to medical conditions (both physical and mental health), claims experience, the receipt of health care, medical history, genetic information, evidence of insurability and disability. Mental health and physical health are the most common aspects to measure one’s health status, and most studies have described health status from the physical perspective [46,47]. Molen and Kocks indicated that health status can closely reflect the direct influence of disease on functional status, symptoms and mental health [48]. Additionally, the health status measure instruction is widely used, such as the SF-36 and other health status questionnaires, containing physical and mental health items [49]. Besides, the objective health status and subjective health status are the two kinds of health status, and the objective health status usually focuses on the objective clinical assessments and the number of chronic medical conditions, but the subjective health status indicates self-reported states of individual health [50]. In this study, we adopted self-rated physical and mental health to measure migrants’ social integration, considering the difficulty in conducting a measurement of the objective health status for the large scale of migrants in China.

The “healthy migrant” paradox implies that migrants may have a better health status than natives when they first came to cities, although they have a lower socioeconomic status [51]. According to the study of Li, Chinese rural–urban migrants experience various kinds of stigmatization in their daily life, which has negative effects on migrants’ health [52]. In the initial period of migration, migrants leave their friends and family and face a new setting where climate, language, housing and food may be different; these all are social stressors. After this period, some other environmental stress such as social support and other institution issues also bother migrants [53]. As mentioned above, these challenges all form migrants’ social stress, which is more closely related to migrants than natives. Social stress from different aspects can damage migrants’ mental health [54]. A study conducted on migrants’ depression in Chengdu city found that migrants’ poor health also contributed to depressive symptoms, and better health helps migrants avoid depressive symptoms [55]. Additionally, migrants to cities are more likely to suffer social discrimination, which also impacts their mental health [56]. As discussed above, there are many health risks for migrants to cities, which may lead to a low degree of social integration. Nevertheless, there is little empirical evidence about whether health status impacts migrants’ social integration, especially in developing countries.

Evidence has indicated that migration experiences may affect migrants’ health, which increases their health costs, which are already a serious challenge in cities [17]. Although migration experiences can also improve mental health in certain conditions, all of this evidence shows that health status is a crucial aspect related to migration [57,58]. Previous studies have found that the mental health of rural–urban migrants is at high risk and requires attention [54,59]. Studies have also shown that rural–urban migrants are more likely to have infectious diseases, which entail great health risks [28]. Additionally, mental health and physical health are both important for migrants, although some studies have focused on only one of these aspects [60,61]. A good health status can facilitate individual income and life satisfaction and even has a potential role in economic development [25,62]. According to the “the salmon basis” hypothesis, migrants with poor health are more likely to return to their country of origin. In this process, we find both that health plays a vital role in migrants’ decisions and that the direct effects of health are significant. Nevertheless, previous studies have mainly focused on the impact of health on migrants’ settlement intention, but the degree of social integration may have a more important function in building a harmonious society and facilitating inclusive urbanization because it is closer to migrants’ subjective perceptions.

Migrants are vulnerable to health risks, which indicates that health issues are too important for migrants to ignore. According to Xie’s research, both physical and mental health status significantly impact migrants’ settlement intention, and we can conclude that mental health and physical health are associated with migrants’ decisions [47]. Although there has been no research on the relationship between settlement intention and social integration, there is no doubt that facilitating social integration may increase migrants’ settlement intentions. Insurance is another important factor linked to health status, and Qin noted that health insurance can help migrants integrate into cities, which has also been proven by many studies [39,63,64,65]. The health of migrants who lack insurance may be endangered, leading to a low social integration level. However, there is no empirical evidence on whether health status impacts social integration and to what extent. As the number of migrants in China will grow rapidly in the future, it is necessary to study the association between migrants’ health and social integration. Therefore, in this study, we focus on the effect of migrants’ health status on their social integration. As the analysis above suggests, we expected health status to impact migrants’ social integration, including their mental and physical health. Additionally, previous studies have noted that mental and physical health usually mutually impact each other, so we hypothesized that they are jointly impacted by the two indicators [66].

Moreover, as we discussed above, social discrimination, social ties and social interactions can all impact migrants’ health during the migration experience, which has been proven by a large number of studies [54,56,67,68,69]. These factors are all tightly associated with social participation among migrants to cities, because most of these are contained in the process of social participation. Additionally, most importantly, the government usually makes efforts to improve social integration in metropolises to improve migrants’ social integration, especially in residential communities. In addition, social participation, especially participation in social activities or organizations, has a significant impact on migrants’ health [70,71,72,73,74]. A meta-analysis including 148 studies indicated that social relationships have an important effect on health status and that social relationships were mainly built through social participation [75]. As mentioned above, social participation may impact migrants’ social ties and social intercourse, so we can expect that the relationship between health status and social integration to be moderated by social participation, which has a clear policy orientation for central and regional governments. In the following sections, large-scale survey data were used to test our hypotheses.

## 4. Research Data, Variables and Methods

### 4.1. Data

The data utilized in this paper were taken from the Chinese Migrants Dynamic Survey (CMDS), which was collected by China’s National Health and Family Planning Commission (NHFPC) in 2014. This dataset focused on migrants in urban regions without a local urban hukou, and this dataset was widely used in research on Chinese migrants. In this dataset, all migrants are aged between 15 and 59 years old and have lived in urban areas for more than one month. The stratified and multistage probability proportionate to size (PPS) sample method was used in this survey to make the dataset more representative. These data from 2014 were chosen based on a sub-survey about “social integration and psychological health” conducted in eight large Chinese cities: Guangzhou city, Zhongshan city, Zhengzhou city, Shenzhen city, Chengdu city, Jiaxing city, Qingdao city and Chaoyang district in Beijing city. The sub-survey not only measures the same basic information as the main questionnaire, but also includes questions about social participation and related questions about migrants’ social sense and integration. To increase the credibility of our study, individuals who responded with default values were excluded before conducting the empirical study, and the final dataset included 12,686 rural–urban migrant individuals and 2037 urban–urban migrant individuals, which were the basic data for further empirical analysis.

### 4.2. Variable

#### 4.2.1. Social Integration

The key independent variable in our study was the degree of migrants’ social integration, which is a crucial aspect of building an inclusive society and is closely related to migrants’ objective well-being. To quantitively measure this variable, we adapted two core indicators to represent social integration: “belonging” and “harmony”. First, belonging was used to describe the migrants’ sense of belonging to the city, which is an important aspect of social integration and has been used in many studies for the measurement of social integration [76]. Usually, migrants may feel they do not belong to the city, although they have lived in cities for a long time, because Chinese migrants tend to have the notion that they are considered as outsiders, so identification is extremely important for determining the degree of social integration [44]. If a person has a strong sense of belonging to the city in which they live and work, we can confirm that the person’s social integration level is high, and Zheng utilized this indicator to measure migrants’ social integration in China [10]. The second indicator, “harmony”, measures the relationship with the surrounding circumstance and residents. Those with a high social integration level may be more likely to have better relationships with others. In addition, harmony is the goal of urban construction in recent rapid urbanization backgrounds, which are tightly associated with social integration, especially in large cities with numerous migrants. Social conflict can also be relieved by increasing “harmony”. A high degree of social integration entails a high level of identification, which is associated with a good personal relationship. Migrants can build good relationships with locals and those in their neighborhoods, which can show that they have successfully integrated into cities and obtained a high degree of social integration. The two indicators we selected were also used to measure social integration by Lin and Dale, as they can comprehensively represent social integration, including a sense of belonging, personal control and generalized trust [77]. The indicators were measured via two representative questions: the first was “Do you agree that you are one of the members of this city?”, and the second was “Do you think you or your family get along with local residents?”. For the first question, the corresponding answers ranged from 1 to 4, representing “Totally disagree”, “Disagree”, “Agree” and “Totally agree”. The answers to the second question ranged from 1 to 5, representing “Very little communication with others”, “Limited communication with others”, “Hard to say”, “Get along occasionally” and “Get along very well”. To avoid including ambiguous answers, the answers were recoded into two categories: “Totally agree” or “Get along very well” and others (1 = “Totally agree” or “Get along very well”; 0 = “Others”).

#### 4.2.2. Physical Health and Mental Health

In our study, two indicators were used to measure the health status of migrants: physical and mental health. Self-rated health has been widely used to calculate individual physical health and is usually rated on a Likert scale. In our study, the quantitative description of physical health was consistent with previous studies. The question about self-rated health was “How would you rate your current state of overall physical health?” The respondents used a 5-point Likert scale, coded as “very poor”, “poor”, “fair”, “good” and “excellent”.

Migrants’ mental health was measured based on a series of related questions surveying the migrants’ unhealthy emotions. According to Xu’s study, we used the same approach to record individuals’ mental health [78]. The six questions included “Did you feel nervous?”, “Did you feel hopeless?”, “Did you feel fidgety?”, “Did you feel so depressed that nothing could cheer up you?”, “Did you feel everything is difficult? ” and “Did you feel that you are worthless”, and the answers were collected using a Likert scale ranging from 1 to 5, representing “never”, “occasionally”, “part of the time”, “most of the time” and “all the time”. In addition, the answers were based on the migrants’ experiences over the last month. Finally, these scores were added together to create a comprehensive variable representing the migrants’ mental health. For convenience and to maintain consistency with physical health, we made this variable a positive index, so a low score indicated poor mental health and vice versa.

#### 4.2.3. Social Participation

To test the moderating effect of social participation in the empirical study, we built a social participation index that contained seven aspects. These aspects were measured by seven questions which focused on migrants’ social activities or organizations, including chambers of commerce, labor unions, voluntary associations, floating party branches, local party branches, fellow-student associations and townsmen associations. Social participation was treated as a dichotomous variable: “1” for participating and “0” for not participating, and the overall score was the sum of these seven items. A higher score represented a higher level of social participation. Additionally, we assumed that the social participation process was stable and persistent for migrant individuals during the period living in the host cities.

#### 4.2.4. Other Control Variables

To improve the accuracy of the model, our study also added series of related control variables in all regression models. Previous studies have provided guidance for control variable selection, which may affect migrants’ social integration [10,79]. In our study, we chose the control variables from individual characteristics, migration factors, socioeconomic status and social support factors. For the individual characteristics, we added the migrants’ age, gender, marital status, education level, employment type and arrangement of children; migration factors included the duration of migration in years and migration distance in terms of “intracity”, “intraprovincial” and “interprovincial” [80]. The socioeconomic status characteristics measured included individual income over the last month, type of housing, workplace and neighborhood characteristics. Lin found that migrants’ social integration levels may vary with neighborhood circumstances [19]. For the social support factor, we chose participating in urban workers’ basic health insurance as the factor representing migrants’ insurance participation status, which has been proven to be a crucial index for migrants’ social integration. In addition, housing type and condition are also important variables that can affect migrants’ social integration which need be added as control variables [10,81].

### 4.3. Statistical Model

For the empirical research, we selected the binary logistic regression model according to our variables’ characteristics. Because the level of social integration was recorded as either “1” (good social integration level) or “0” (others), a traditional multiple linear regression was less suitable than the logistic model. The logistic model can reflect how ceteris paribus changes in the independent variables influence the independent variable, which was the degree of social integration in our research. The probability function of the logistic model is shown below:

(1) $$P=\frac{\exp(Z)}{1+\exp(Z)}$$

(2) $$Z=b_{0}+b_{1}x_{1}+b_{2}x_{2}+b_{3}x_{3}+,...,b_{n}x_{i}+\varepsilon_{i}=b_{0}+\overset{n}{\underset{i=1}{\sum }}b_{i}x_{i}$$

where *Ρ* is the probability of migrants having a good social integration level. *Z* in Equation (2) is the linear combination of $x_{i}\left(i=1,\;2,\;3,\ldots ,n\right)$, which is the variable affecting migrants’ social integration, and $b_{i}\left(i=1,2,3,\ldots ,n\right)$ is the regression coefficient, which can represent the contribution of $x_{i}$ to *Ρ*. *n* is the number of observations, *b*_0_ is the constant term and Equation (1) is the response probability. The regression was carried out using a maximum likelihood estimation (MLE). Additionally, city-fixed effects were controlled when running the regression models because the data were collected from eight cities.

## 5. Results

### 5.1. Descriptive Statistics

The characteristics of the variables we chose are shown in Table 1. In our data, only 34.49% of the migrants had high belonging values, and we further analyzed the value across different migrant groups. We found that fewer rural–urban migrants (33.58%) had high belonging values than urban–urban migrants (40.16%), although both values were low. The harmony values were also relatively low: only 28.94% of the migrants reported getting along well with local residents, with rates of 28.04% and 34.56% among rural–urban and urban–urban migrants, respectively, which means that the relationship between migrants and local residents can be greatly improved, especially among rural–urban migrants. Therefore, we can conclude that the migrants’ social integration was at a low level, especially among rural–urban migrants. The descriptive statistics revealed that the mean physical health status value was 3.77, which was at an above-average level between “fair” and “good”, and that 38.16% of the migrants had below-average physical health. The mental health score of the migrants was 26.57, indicating that their mental health was not poor, and approximately 42.29% of the migrants reported values below this level. Therefore, we can conclude that the migrants’ health was not at a satisfactory level and needs more attention.

With respect to individual characteristics, there were more male migrants than female migrants, which indicates that males prefer to move to large cities for job opportunities and higher incomes in urban China. The mean income of the migrants was 3694 yuan; however, approximately 65% of migrants reported values below this level. Insurance participation can promote migrants’ social integration according to a previous study, but approximately 70% of the migrants did not have insurance, which is a serious issue for our society. Additionally, having no stable residence was another important feature for migrants reported by approximately 80% of all migrants. The average age of migrants was 32.66, with an average migration length of 4.22 years in host cities.

### 5.2. Basic Regression Results

The results of the basic regression model using logistic regression are shown in Table 2. In model 0, we added all the control variables for comparison with other models (for belonging index). Next, we added physical health and mental health to models 1 to 6 (1–3 for belonging and 4–6 for harmony). The results of the regression model were stable when we changed the variable combination, which shows the robustness of our model.

First, all effects of health status, including physical health and mental health, were statistically significant at the 1% level and positive on the representative social integration index (belonging and harmony), as expected. This indicates that migrants who have better physical and mental health are more likely to have a higher degree of social integration, including a higher sense of belonging and a harmonious relationship with others. Specifically, a one-point increase in physical and mental health will improve the odds of good social integration by 33.27% and 5.98% for belonging and 66.05% and 6.35% for harmony, respectively. Our findings show that physical health status plays a more important role than mental health status for migrants’ social integration, although both health statuses significantly impact migrants’ social integration. Additionally, a series of individual control variables, such as employment category, individual income and length of migration, were significantly related to the level of social integration. Migrants who have native neighborhoods, live in a stable home and participate in social insurance may have higher social integration levels. Intercity migration, which represents near-distance migration, may lead to a higher social integration level than migrations of greater distance, consistent with previous studies. The reason shorter migration distances result in higher social integration is that such migrations entail fewer challenges, such as differences in dialects and customs [21,82]. Additionally, the study yielded some interesting findings; for example, migrants, regardless of rural–urban or urban–urban migrants, may have a higher level of social integration when their children accompany them to the host cities.

### 5.3. Heterogeneity Analysis

There are two groups of migrants in China: rural–urban migrants and urban–urban migrants. However, these two groups have many differences, and the biggest is that urban–urban migrants have an urban hukou and have lived in urban regions before. Rural–urban migrants have no urban hukou and have previously lived in rural regions, unlike urban migrants. Of the two groups, rural–urban migrants are in a more serious marginal position. It is of great significance to distinguish these two groups and analyze their heterogeneity in terms of health to enable more precise policies. For this purpose, we divided the 14,723 migrants into two groups, according to their hukou status. After separating the migrants, there were 12,686 rural–urban migrants and 2037 urban–urban migrants, which were added to our logistic model for further analysis. Before that, we compared their social integration levels using their mean values of the degree of social integration. The mean values of belonging for rural–urban and urban–urban migrants were 0.34 and 0.41, respectively, and the harmony values were 0.28 and 0.35, respectively. We found that both social integration indexes were obviously lower among rural–urban migrants than among urban–urban migrants because urban–urban migrants have lived and worked in urban regions before their current migration experiences, and they are more familiar with city environments. However, rural–urban migrants may face a new environment, which becomes a huge hurdle to integration.

The results of the heterogeneity analysis are shown in Table 3. The impacts of health on the degree of the two social integration indexes were consistent, positive and significant. Additionally, the effect of health on social integration was robust for both migrant groups, which indicates the robustness of the conclusion obtained in the last section. However, the coefficients of physical and mental health showed different effects on belonging and harmony. Comparing these four models, in general, physical and mental health were positively and significantly correlated with social integration indexes, which indicates that the two health status indicators are equally important for the two migrant groups; in other words, the effect of health does not change across migrant groups. Nevertheless, the effect of physical health was relatively more important than that of mental health for migrants’ social integration. Specifically, the effect of physical health on harmony was more important than that on belonging, but the effect of mental health on the two indicators was relatively consistent. In summary, the effect of health status on social integration level is consistent with the regression results in the last section, which further supports our conclusion.

There were also meaningful findings for other factors. For the two migrant groups, although the effect of health was stable and consistent, some variables revealed interesting changes that require more attention. For example, rural–urban migrants’ social integration was more sensitive to employment features, individual income, neighborhood features and housing conditions, which means that policies need to be designed for specific groups according to their needs for social integration.

### 5.4. Moderate Effect Analysis

To test whether social participation moderated the effect of health status on social integration, a stepwise strategy was used. Several interaction items were added into the regression model to avoid multicollinearity. The results are shown in Table 4. As the results show, all the key independent variables in these six models were consistent with the basic regression model, supporting the robustness of our conclusion. Compared with the coefficients of the intersections, social participation moderated the effect of migrants’ mental health on social integration; in other words, migrants’ social participation can strengthen the effect of mental health status on social integration. However, it cannot moderate the effect of physical health on the social integration level. In Model 3 and Model 6, the coefficients of the interaction term were significant, which suggests that social integration is jointly determined by the two health indicators and verifies the hypothesis above. Therefore, we can conclude that social participation is crucial for migrants’ social integration, which has a potential pathway to facilitate the social integration of marginalized populations.

### 5.5. Robustness Analysis

To test our research’s robustness, in this section, according to previous studies, we chose three common methods to do this. For example, replacing the key independent variable of the model was the most commonly used method. First, health status, including physical health and mental health, could be summarized by one indicator using the question “Do you agree that you are more likely to get sick than others”, with the possible answers of “Yes” and “No”. Therefore, we built another indicator to represent migrants’ health status, and this indicator was tightly associated with physical and mental health. We replaced the two health indicators in our study with the new indicator and ran the basic logistic regression model again; the regression results are shown in Table 5. As the table displays, the regression results were consistent with the basic regression in Table 1. Health indicators have a significant and positive effect on the two aspects of social integration, which indicates our results’ robustness.

Second, apart from the logistic regression model, we adopted the OLS regression model, which can indicate robustness. Therefore, to utilize the OLS model, the independent variables were not divided into binary values, as in the logistic model. The social integration values, including the values for belonging and harmony, all ranged from 1–4 and 1–5, respectively, and the other key dependent variables and control variables were the same as those described above. The OLS regression results are also shown in Table 5. We found that the two key health status variables were also significant and positive, consistent with the results of basic model. Therefore, we can conclude that the results were still robust after changing the regression model.

Third, according to previous studies, dividing samples before running regression models is useful for completing the test. As far as we know, it is a natural law that young people are more likely to have better health than elderly people. However, in our dataset, the range of the migrants’ ages was 18–60, which is very large. Therefore, the health statuses of the participants might have differed sharply, and we divided the dataset based on migrant age. According to the World Health Organization (WHO), adults younger than 44 years of age are considered young, and those between 44 and 59 years of age are considered middle-aged. We ran the regression model on the two groups of migrants (dividing by 44 years old). The results of the regression are presented in Table 5 below. We can see that all coefficients of physical and mental health were positive and significant at the 1% level for both the young and middle-aged groups, which proves that our results are robust. In summary, our results are robust, as proven through three robust test methods, and we can conclude that health status, including physical health and mental health, has a significant effect on migrants’ social integration.

## 6. Discussion

Although some studies have attempted to identify the determinants of migrants’ social integration in cities, few studies have taken migrants’ health into consideration. Our study explored the impact of migrants’ health status on their social integration level. Based on classical theories and previous related studies, we proposed our hypothesis about the impact of health on social integration and utilized a large-scale migrant survey dataset to test our hypothesis. The main goal of our study was to provide insights into the determinants of migrants’ social integration level and facilitate the construction of an inclusive society.

The results of this paper show that migrants’ health is a vital factor in their social integration in terms of both their sense of belonging to cities and their harmonious relationship with their surroundings. Our results provide empirical evidence on the effects of migrants’ health at the social integration level, which is a new pathway to improve migrants’ social integration that has not been widely investigated before. Our results are similar to those of some studies focusing on migrants’ intention to settle in cities, which indicated that better health status may lead to higher settlement intention and that settlement intention is impacted by many factors. Because social integration is associated with settlement intention, a low social integration level may influence migrants to return to their hometowns. If migrants are in poor health, on the one hand, it will impact their individual income and increase the cost of medicine or medical services; thus, low economic status or economic integration is an important aspect for migrants. On the other hand, poor health leads to less interaction with one’s surroundings, which decreases one’s social integration level. Therefore, when a migrant is in poor health and lacks high-quality public healthcare, he or she may easily feel a low degree of social integration.

Moreover, our results indicate that migrants with better health, including physical and mental health, may have a higher social integration level and be jointly impacted by the two indicators. One of the most important findings of the current study is that the effect of health is equally important for rural–urban and urban–urban migrants. This finding highlights that health status plays a crucial role in the social integration of migrants of both types. This finding has a clear policy implication for facilitating migrants’ social integration level, which requires that the government solve the challenge of low social integration. Another important finding is that social participation can significantly moderate the effect of mental health on the social integration level and cannot moderate the effect of physical health. As indicated in previous studies, social discrimination was the main factor impacting migrants’ mental health and indirectly impacting migrants’ social integration [9,56,68,69]. This may be because when migrants have a higher social participation level and relatively low mental health status, their social integration level is increasingly dependent on social contact during social activities or in organizations. Social participation, as a process of intercourse, can efficiently moderate the effect of health on migrants’ social integration level. However, we indicated that the moderate function of social participation is more significant for mental health, which means our government needs to provide more opportunities for migrants to improve their physical health and the quality of healthcare. Additionally, in the robustness test section, we also found that health is a crucial factor in social integration across ages, which highlights the importance of health for migrants.

Our results have important theoretical implications for inner migrant research, because previous studies, when discussing the factors impacting social integration, have generally ignored migrants’ health issues. In this study, both physical and mental health were discussed to provide a comprehensive understanding, which has important implications not only for related research, but also for our government. Traditional theories usually consider health to be an important factor in migrants’ intentions to settle but ignore the effect on social integration level. This study emphasizes the effect of health on social integration and clarifies the mechanism of social participation in this process.

Our findings have several policy implications for improving migrants’ social integration levels in the future. First, migrants are marginalized in cities, do not have a local hukou, face substantial health risks and have health vulnerabilities due to low-quality health services; thus, city governments should initially pay more attention to health services and improve the working and living environments of migrants. However, in some metropolises, the hukou system has been shown to be resistant to quick reform, and the government should implement creative policies for these migrants; for example, migrants with a rural hukou usually access medical services in rural areas, so the government can design an institution for them to transfer their rural medical insurance to urban medical insurance or a special insurance policy for migrants to cites. For living and working environments, the government needs to provide more affordable houses, especially for migrants. Additionally, to improve migrants’ physical and mental health, policymakers should take migrants’ physical and mental health care into consideration when discussing the pathway of inclusive urbanization in the future. The government should ensure related services, such as psychological counselling agencies, to monitor and treat migrants’ physical and mental issues. Second, social participation can play a positive role in the process by which health status impacts social integration; however, migrants, especially those with a poor economic status, may live in urban villages and have limited opportunities to participate in social activities and organizations. It is difficult for them to build social ties on their own. The government should help them join social organizations and activities. For example, in some settlements or communities with large-scale migration, the government or city administrators should enable migrants to join local activities or organizations and design activities to include migrants. Third, we found that migrants with children and undergoing short-distance migration can have a higher social integration level; however, in some metropolises, it is difficult for migrants’ children to obtain high-quality education without a high cost [33,83]. Considering the limited pathway for their children to obtain education and the high cost, migrants usually leave their children in their hometowns, which not only harms the children’s physical and mental health, but also may impact migrants’ social integration in their new cities due to worries about their children and loneliness [84]. Policy designers should take this finding into consideration and provide low-cost education access for migrants’ children. For the question that long-distance migration may lead to a low social integration level, within the concentrated populations in main urban regions, to avoid long-distance migration, more work opportunities should be provided in regional centers, especially in undeveloped provinces, which can give migrants a more familiar living environment with which to adapt.

Briefly, in previous studies, institutional barriers were widely discussed as a main factor impacting migrants’ social integration. This paper emphasizes the impact of health on social integration, which is more related to the individual level and should not be ignored when discussing migrants’ social integration. In the future, more migrants will move to cities for jobs and better lives, and the government should take actions to implement more targeted policies, not only protecting migrants’ physical and mental safety, but also ensuring inclusive urbanization.

## 7. Conclusions

By utilizing the Chinese Migrant Dynamic Survey dataset and logistic regression model, this study investigated the association between health status, including physical and mental health, and the degree of social integration in Chinese metropolises, and it also explored the impact among rural–urban and urban–urban migrants, which has rarely been studied before. Our results indicate that both physical and mental health status are vital to migrants’ social integration level. The effect of health status is moderated by migrants’ social participation. Additionally, we also found that the impacts of both mental and physical health on social integration level are consistent, significant and positive among rural–urban and urban–urban migrants. Based on our results, we highlight the significance of health status, whether physical or mental health, which is vital for migrants to cities due to the high level of social integration. Therefore, we can conclude that improving migrants’ health status can significantly facilitate their social integration level and make them feel that they belong to society and better adapt to the living conditions of their new cities. Migrants, especially rural–urban migrants, usually face difficult working and living environments that lack sufficient health care and need the government to pay more attention to their health status and to implement countermeasures to solve these problems, such as upgrading the healthcare system for people who have no local hukou, breaking the hukou system in more cities and improving the health status monitoring institutions for migrants. Moreover, social participation has been proven to mediate the impact of mental health on social integration. Migrants are discriminated against in cities because of their marginalized positions, and participating in social activities and organizations can be hard for them. Therefore, local governments should implement more targeted policies to push migrants to join social activities and build tight social relationships to improve their degree of social integration. In brief, our study highlights the importance of health status on migrants’ social integration, which is a new pathway for addressing migrants’ social integration problems for a “people-centered” society. Meanwhile, we explored the mechanism by which health affects social integration and found that social participation plays a key role in this process for mental health.

Although we explored the impact of health on migrants’ social integration and showed the importance of migrant health from a new perspective, there were also several limitations to this study. First, we used self-rated health status in our study rather than the objective indicators due to the difficulty of data acquisition, which may have biased the estimation of the migrants’ real health status. Second, this large-scale survey of more than 10,000 migrants focusing on social integration was conducted in 2014, so the findings may not be generalized to more recent migration events. However, in 2014, there was already a large number of migrants in Chinese cities, and the hukou system still exists in metropolises; thus, the study design can be feasibly applied in today or in the future if the hukou system is not eased in metropolises.

## Figures and Tables

**Table 1:** Descriptive statistics.

**Variables**	**Description**	**Frequency**	**Percentage**
Belonging	High sense of belonging	5078	34.49%
	Others	9645	65.51%
Harmony	Get along very well	4261	28.94%
	Others	10,462	71.06%
Gender	Male	8506	57.77%
	Female	6217	42.23%
Occupation	Self-employment	4382	29.76%
	Others	10,341	70.24%
Individual income	Less than mean income	9423	64.00%
(Mean income = 3694 yuan)	More than mean income	5300	36.00%
Insurance	Have urban worker insurance	4545	30.87%
	Others	10,178	69.13%
Work place	Inner city	7280	49.45%
	Others	7443	50.55%
Neighborhood	Local residents	11,659	79.19%
	Others	3064	20.81%
House	Have stable residence (including public policy and own house)	3071	20.86%
	No stable residence	11,652	79.14%
Education	Middle school and below	8800	59.77%
	High school	3701	25.14%
	College and above	2222	15.09%
Migration type	Trans-provincial migration	8016	54.45%
	Interprovince migration	6146	41.74%
	Intercity migration	561	3.81%
**Continuous variables**	**Description**	**Mean**	**(S.D)**
Physical health	Physical health level	3.77	0.97
Mental health score	Total score	26.57	3.06
Age	Age	32.66	8.67
Length	Migration time	4.22	4.39

**Table 2:** Logistic regression results on the effects of physical and mental health of migrants.

		Belonging			Harmony		
	Model 0	Model 1	Model 2	Model 3	Model 4	Model 5	Model 6
Constant	−1.5237 *** (0.0987)	−2.9023 *** (0.1324)	−3.4512 *** (0.1967)	−4.3109 *** (0.2075)	−3.9848 *** (0.1440)	−3.9059 *** (0.2109)	−5.4912 *** (0.2269)
Self-employed (ref. others)	0.1127 ** (0.0445)	0.1006 * (0.0449)	0.1043 ** (0.0448)	0.0949 * (0.0451)	0.1451 *** (0.0478)	0.1516 *** (0.0669)	0.1391 *** (0.0479)
individual income (ref. Average income and below)	0.1037 *** (0.0403)	0.0947 ** (0.0406)	0.1021 ** (0.0405)	0.0946 * (0.0408)	0.1432 *** (0.0432)	0.1541 *** (0.0425)	0.1432 *** (0.0433)
Length	0.0216 *** (0.0045)	0.0214 *** (0.0045)	0.0207 *** (0.0045)	0.0206 *** (0.0045)	0.0223 *** (0.0048)	0.0212 *** (0.0047)	0.0215 *** (0.0048)
Job place (ref. out of urban)	−0.0015 (0.0384)	0.0236 (0.0388)	0.0020 (0.0386)	0.0239 (0.0389)	−0.1694 *** (0.0413)	−0.2055 *** (0.0405)	−0.1719 *** (0.0414)
Age	−0.0018 (0.0024)	0.0035 (0.0025)	−0.0025 (0.0024)	0.0025 (0.0025)	0.0140 *** (0.0026)	0.0040 (0.0025)	0.0129 *** (0.0026)
Neighborhood (ref. migrants)	0.2879 *** (0.0450)	0.2564 *** (0.0455)	0.2798 *** (0.0453)	0.2535 *** (0.0457)	0.4076 *** (0.0471)	0.4393 *** (0.0462)	0.4052 *** (0.0473)
Gender (ref. male)	−0.0351 (0.0372)	−0.0774 ** (0.0376)	−0.0411 (0.0374)	−0.0782 ** (0.0377)	−0.1670 *** (0.0401)	−0.1000 ** (0.0393)	−0.1682 *** (0.0401)
House (ref. no stable house)	0.3011 *** (0.0440)	0.2860 *** (0.0445)	0.2927 *** (0.0443)	0.2806 *** (0.0446)	0.1776 *** (0.0472)	0.1923 *** (0.0462)	0.1708 *** (0.0474)
Education (ref. primary school and below)							
Middle school	0.0689 (0.0452)	0.0494 (0.0456)	0.0791 * (0.0454)	0.0581 (0.0458)	0.0712 (0.0484)	0.1122 ** (0.0479)	0.0777 (0.0490)
High school and above	0.0989 (0.0605)	0.0876 (0.0611)	0.1165 * (0.0609)	0.1013 * (0.0613)	0.1659 ** (0.0646)	0.1989 *** (0.0636)	0.1782 *** (0.0649)
Insurance (ref.no insurance)	0.2151 *** (0.0439)	0.2093 *** (0.0044)	0.2191 *** (0.0441)	0.2052 *** (0.0445)	0.3104 *** (0.0472)	0.3088 *** (0.0463)	0.3085 *** (0.0474)
Arrangement of Children (ref. Migrant with child)	0.3044 *** (0.0404)	0.3020 *** (0.0408)	0.2953 *** (0.0406)	0.2951 *** (0.0409)	0.1946 *** (0.0434)	0.1876 *** (0.0427)	0.1865 *** (0.0436)
flow type (ref. Intro-province migration)							
Inter-province migration	0.0695 (0.0452)	0.0720 (0.0492)	0.0668 (0.0454)	0.0693 (0.0493)	0.2906 *** (0.0527)	0.2760 *** (0.0519)	0.2887 *** (0.0529)
intercity migration	0.4734 *** (0.0993)	0.4671 *** (0.1002)	0.4697 *** (0.0998)	0.4635 *** (0.1005)	0.6997 *** (0.1033)	0.6882 *** (0.1013)	0.6962 *** (0.1036)
Physical health		0.3157 *** (0.0196)		0.2872 *** (0.0200)	0.5349 *** (0.0217)		0.5071 *** (0.0220)
Mental health			0.0732 *** (0.0064)	0.0581 *** (0.0065)		0.0869 *** (0.0069)	0.0616 *** (0.0070)
region-fixed effects	YES	YES	YES	YES	YES	YES	YES
Observations	14,723	14,723	14,723	14,723	14,723	14,723	14,723
Prob > chi2	0.0000	0.0000	0.0000	0.0000	0.0000	0.0000	0.0000
Pseudo R^2^	0.0464	0.0604	0.0537	0.0649	0.0889	0.0614	0.0934

Note: * represents *p* < 0.1. ** represents *p* < 0.05. *** represents *p* < 0.01.

**Table 3:** Logistic regression results on rural–urban and urban–urban migrants.

	Rural–Urban Migrants		Urban–Urban Migrants	
	Model 1	Model 2	Model 3	Model 4
	Belonging	Harmony	Belonging	Harmony
Constant	−4.2695 *** (0.2282)	−5.5597 *** (0.2502)	−4.7405 *** (0.5300)	−4.9708 *** (0.5630)
Self-employed (ref. others)	0.0863 *** (0.0485)	0.1488 *** (0.0516)	0.1213 (0.1258)	0.0639 (0.1317)
individual income	0.1260 *** (0.0442)	0.1341 *** (0.0471)	−0.1212 (0.1094)	0.2015 * (0.1147)
(ref. Average income and below)				
Length	0.0171 *** (0.0049)	0.0227 *** (0.0052)	0.0423 *** (0.0118)	0.0140 (0.0123)
Job place (ref. out of urban)	0.0258 (0.0422)	−0.1778 *** (0.0451)	0.0224 (0.1050)	−0.1526 (0.1089)
Age	0.0001 (0.0024)	0.0132 *** (0.0028)	0.0151 ** (0.0071)	0.0114 (0.0075)
Neighborhood (ref. migrants)	0.2768 *** (0.0498)	0.4459 *** (0.0516)	0.1128 (0.1164)	0.1829 (0.1189)
Gender (ref. male)	−0.0704 * (0.0409)	−0.1472 *** (0.0437)	−0.1235 (0.0997)	−0.2880 *** (0.1039)
House (ref. no stable house)	0.2977 *** (0.0497)	0.1640 *** (0.0530)	0.1383 (0.1058)	0.1900 * (0.1092)
Education				
(ref. primary school and below)				
Middle school	0.0576 (0.0489)	0.0963 * (0.0523)	0.0928 (0.1403)	−0.0254 (0.1479)
High school and above	0.0561 (0.0713)	0.1526 ** (0.0754)	0.3079 ** (0.1448)	0.1967 (0.1514)
Insurance (ref. no insurance)	0.2031 *** (0.0490)	0.3210 *** (0.0523)	0.1815 (0.1119)	0.2518 ** (0.1170)
Children place	0.3022 *** (0.0445)	0.1779 *** (0.0474)	0.2760 *** (0.1073)	0.2361 ** (0.1123)
Migration type				
(ref. Trans-provincial migration)				
Interprovince migration	0.0009 (0.0540)	0.2667 *** (0.0581)	0.4048 *** (0.1266)	0.3932 *** (0.1331)
intercity migration	0.4425 *** (0.1119)	0.6603 *** (0.1157)	0.6278 *** (0.2312)	0.8251 *** (0.2365)
Physical health	0.2824 *** (0.0217)	0.5113 *** (0.0240)	0.3375 *** (0.0527)	0.4915 *** (0.0561)
Mental health	0.0574 *** (0.0071)	0.0627 *** (0.0077)	0.0623 *** (0.0160)	0.0556 *** (0.0170)
region-fixed effects	YES	YES	YES	YES
Observations	12,686	12,686	2037	2037
Prob > chi2	0.0000	0.0000	0.0000	0.0000
Pseudo R^2^	0.0635	0.0922	0.0741	0.0928

Note: * represents *p* < 0.1. ** represents *p* < 0.05. *** represents *p* < 0.01.

**Table 4:** Results of moderate effect analysis.

	Belonging			Harmony		
	Model 1	Model 2	Model 3	Model 4	Model 5	Model 6
Constant	−4.2693 *** (0.2281)	−4.2815 *** (0.2284)	−4.3148 *** (0.0059)	−5.5618 *** (0.2503)	−5.5681 *** (0.2502)	−5.4943 *** (0.2468)
Self-employed (ref. others)	0.0862 * (0.0485)	0.0876 * (0.0485)	0.0870 * (0.0485)	0.1493 *** (0.0516)	0.1502 *** (0.0516)	0.1502 *** (0.0516)
individual income (ref. Average income and below)	0.1259 *** (0.0442)	0.1257 *** (0.0442)	0.1245 *** (0.0442)	0.1351 *** (0.0471)	0.1336 *** (0.0471)	0.1327 *** (0.0471)
Length	0.0171 *** (0.0049)	0.0170 *** (0.0049)	0.0169 *** (0.0049)	0.0227 *** (0.0052)	0.0226 *** (0.0052)	0.0225 *** (0.0052)
Job place (ref. Out of urban)	0.0258 (0.0422)	0.0244 (0.0422)	0.0252 (0.0422)	−0.1781 *** (0.0451)	−0.1793 *** (0.0451)	−0.1781 *** (0.0451)
Age	0.0009 (0.0026)	0.0008 (0.0026)	0.0009 (0.0026)	0.0132 *** (0.0028)	0.0131 *** (0.0028)	0.0132 *** (0.0028)
Neighborhood (ref. migrants)	0.2766 *** (0.0498)	0.2793 *** (0.0498)	0.2784 *** (0.0499)	0.4466 *** (0.0517)	0.4481 *** (0.0517)	0.4478 *** (0.0517)
Gender (ref. male)	−0.0702 * (0.0409)	−0.0700 * (0.0409)	−0.0708 * (0.0409)	−0.1476 *** (0.0437)	−0.1470 *** (0.0437)	−0.1471 *** (0.0437)
House (ref. no stable house)	0.2982 *** (0.0497)	0.2953 *** (0.0497)	0.2983 *** (0.0497)	0.1606 *** (0.0530)	0.1610*** (0.0530)	0.1650 *** (0.0530)
Education (ref. Primary school and below)						
Middle school	0.0577 (0.0489)	0.0593 (0.4889)	0.0509 (0.0489)	0.0953 * (0.0524)	0.0980 * (0.0524)	0.0910 * (0.0524)
High school and above	0.0565 (0.0713)	0.0592 (0.0713)	0.0498 (0.0713)	0.1500 ** (0.0755)	0.1555 ** (0.0755)	0.1480 ** (0.0755)
Insurance (ref.no insurance)	0.2032 *** (0.0490)	0.2031 *** (0.0490)	0.2054 *** (0.0491)	0.3195 *** (0.0523)	0.3207 *** (0.0523)	0.3231 *** (0.0523)
Children place	0.3022 *** (0.0445)	0.3009 *** (0.0445)	0.3028 *** (0.0445)	0.1781 *** (0.0474)	0.1762 *** (0.0471)	0.1786 *** (0.0474)
Migration type (ref. Intra-provincial migration)						
Interprovince migration	0.0005 (0.0540)	0.0007 (0.0540)	−0.0023 (0.0541)	0.2689 *** (0.0582)	0.2670 *** (0.0582)	0.2648 *** (0.0582)
intercity migration	0.4415 *** (0.1120)	0.4429 *** (0.1119)	0.4407 *** (0.1120)	0.6666 *** (0.1159)	0.6615 *** (0.1158)	0.6598 *** (0.1158)
Physical health	0.2823 *** (0.0217)	0.2824 *** (0.0217)	0.2836 *** (0.0217)	0.5113 *** (0.0240)	0.5113 *** (0.0240)	0.5095 *** (0.0240)
Mental health	0.0574 *** (0.0071)	0.0581 *** (0.0071)	0.0580 *** (0.0071)	0.0629 *** (0.0077)	0.0632 *** (0.0077)	0.0598 *** (0.0077)
Physical × Social participation	−0.0059 (0.0183)			0.0351 * (0.0200)		
Mental × Social participation		0.0157 ** (0.0062)			0.0160 ** (0.0067)	
Physical × Mental			0.0293 *** (0.0068)			0.0244 *** (0.0077)
region-fixed effects	YES	YES	YES	YES	YES	YES
Observations	12,686	12,686	12,686	12,686	12,686	12,686
Prob > chi2	0.0000	0.0000	0.0000	0.0000	0.0000	0.0000
Pseudo R^2^	0.0635	0.0639	0.0646	0.0924	0.0926	0.0929

Note: * represents *p* < 0.1. ** represents *p* < 0.05. *** represents *p* < 0.01.

**Table 5:** Results of robust test.

	Method 1		Method 2		Method 3 (1 = Young Person; 2 = Middle-Aged Person)			
	Belonging	Harmony	Belonging	Harmony	Belonging 1	Harmony 1	Belonging 2	Harmony 2
Physical health			0.0591 *** (0.0040)	0.0912 *** (0.0038)	0.2930 *** (0.0216)	0.4993 *** (0.0240)	0.2517 *** (0.0522)	0.5420 *** (0.0562)
Mental health			0.0111 *** (0.0013)	0.0100 *** (0.0012)	0.0550 *** (0.0069)	0.0672 *** (0.0076)	0.0809 *** (0.0195)	0.0223 (0.0196)
Replace health status variable	0.2454 *** (0.0186)	0.2757 *** (0.0198)						
Other control variables	Fixed	Fixed	Fixed	Fixed	Fixed	Fixed	Fixed	Fixed
Observations	14,723	14,723	14,723	14,723	12,799	12,799	1924	1924
Prob > chi2	0.0000	0.0000	-	-	0.0637	0.0951	0.0803	0.0976
Pseudo R^2^ (Adj R-squared)	0.0558	0.0631	0.0798	0.1056	-	-	-	-

Note: * represents *p* < 0.1. ** represents *p* < 0.05. *** represents *p* < 0.01.

## Data Availability

The datasets used and analyzed in this study are available from the corresponding author on reasonable request.
